# Identification of Novel p53 Pathway Activating Small-Molecule Compounds Reveals Unexpected Similarities with Known Therapeutic Agents

**DOI:** 10.1371/journal.pone.0012996

**Published:** 2010-09-27

**Authors:** Karita Peltonen, Laureen Colis, Hester Liu, Sari Jäämaa, Henna M. Moore, Juulia Enbäck, Pirjo Laakkonen, Anne Vaahtokari, Richard J. Jones, Taija M. af Hällström, Marikki Laiho

**Affiliations:** 1 Molecular Cancer Biology Program and Department of Virology, Haartman Institute, University of Helsinki, Helsinki, Finland; 2 Sidney Kimmel Comprehensive Cancer Center, Johns Hopkins University, Baltimore, Maryland, United States of America; 3 Laboratory Division, University of Helsinki, Helsinki, Finland; 4 Molecular Cancer Biology Program and Institute of Biomedicine, University of Helsinki, Helsinki, Finland; 5 Molecular Imaging Unit, Biomedicum Helsinki, University of Helsinki, Helsinki, Finland; Bauer Research Foundation, United States of America

## Abstract

Manipulation of the activity of the p53 tumor suppressor pathway has demonstrated potential benefit in preclinical mouse tumor models and has entered human clinical trials. We describe here an improved, extensive small-molecule chemical compound library screen for p53 pathway activation in a human cancer cell line devised to identify hits with potent antitumor activity. We uncover six novel small-molecule lead compounds, which activate p53 and repress the growth of human cancer cells. Two tested compounds suppress *in vivo* tumor growth in an orthotopic mouse model of human B-cell lymphoma. All compounds interact with DNA, and two activate p53 pathway in a DNA damage signaling-dependent manner. A further screen of a drug library of approved drugs for medicinal uses and analysis of gene-expression signatures of the novel compounds revealed similarities to known DNA intercalating and topoisomerase interfering agents and unexpected connectivities to known drugs without previously demonstrated anticancer activities. These included several neuroleptics, glycosides, antihistamines and adrenoreceptor antagonists. This unbiased screen pinpoints interference with the DNA topology as the predominant mean of pharmacological activation of the p53 pathway and identifies potential novel antitumor agents.

## Introduction

p53 is a key activator of cellular cascades governing cell life and death [1], [2]. It is activated in response to both physiological and non-physiological stresses such as oxidative, viral, oncogenic and genotoxic stress, and hypoxia [1]–[3]. During tumor evolution, the p53 gene, *TP53*, is frequently mutated, yet, over 50% of human tumors harbor the wild type gene [3], [4]. The p53 pathway has been an attractive target for therapeutic manipulation. It has been proposed that the activation of p53 pathway enhances tumor cell killing [1]–[3], [5]. Genetic evidence, both in human and mice, definitively show the essential tumor suppressive activity of *TP53* [4], [6], [7]. More recent, switchable p53 expression models in mouse demonstrate that activation of p53 expression leads to regression of several tumor types by invoking apoptosis, senescence and the cellular innate immunity [8]–[10].

The current p53-related experimental therapeutic arsenal can be classified to those with known mechanisms of action (*e.g.* Hdm2 inhibitors) and to drugs that have demonstrated activation of either wild type (wt) or mutant p53 cells but no or poorly understood mechanisms of action. These have arisen through targeted drug design (Hdm2-inhibitors like nutlin-3 and MI-219) or through screens for wt and mutant p53 activating small-molecule compounds [11]–[19]. Pilot studies using Hdm2 inhibitory compounds show remarkable in vivo anti-tumor effects without side effects [13], [17], [19], [20]. We have established that nutlin-3 is the first and highly effective agent inducing B-cell lymphoma (Kaposi's sarcoma herpes virus (KSHV) infected pleural effusion lymphoma) cell killing both in vitro and in vivo mouse models [20]. Thus, based on these studies, inactivation of the p53 pathway by the KSHV virus lies in the pathogenesis of this incurable malignancy. Furthermore, these studies provide an indication that depending on the context (*i.e.* genetic composition and inherent dysfunctional pathways) of the tumor, activation of the p53 pathway can launch a cytotoxic response.

Mechanisms of action of the p53 pathway affecting drugs, with the exception of the Hdm2 inhibitors, are largely unresolved [19]. p53, structurally, is not easily amenable for targeting by small-molecule compounds. Many of the drugs identified to activate either wild-type or mutant p53 function arose from screening protocols using p53 sequence-specific binding and consequent reporter activation [19]. These unbiased screens have likely yielded compounds acting upstream of p53 to provoke p53 activation. This is illustrated by the fact that many of the identified drugs have also p53 independent functions to suppress tumor cell growth. Encouragingly, several of the compounds activate p53 without launching a cellular DNA damage response (*i.e.* do not impose genotoxic effects on the normal tissues), indicating that they employ activation of events other than DNA damage [19].

Given that p53 is a key activator of cell cycle arrest/apoptosis pathways, we considered that further development of small-molecule compounds inducing p53 is highly warranted and has potential for therapeutic exploitation. We demonstrate here successful implementation of a cell-based high-content imaging screen to identify novel p53 pathway activating small-molecule lead compounds. This screen was devised to identify hits even in the presence of activation of the powerful p53-mediated apoptotic pathway. We present, both by genomic profiling and screening of a defined drug library, that the principal mean to activate p53 pathway is related to interference with DNA topology, likely by DNA intercalation. These approaches identify a substantial number of both new experimental lead molecules and drugs with well-known pharmacological profiles as potentially useful anticancer compounds.

## Materials and Methods

### p53 activity screen

A cell-based assay for p53-dependent expression of a fluorescent reporter was established. A375 melanoma cells (ATCC CRL-1619) were stably transfected with a DsRed Express (Clontech) vector containing p53 consensus element [21] (p53DsRed reporter). p53DsRed reporter activation was verified by UVC, ionizing radiation (^137^Cs source) and nutlin-3 (Alexis Biochemicals) treatments.

Cells were plated at 10,000 cells/well onto 96-well plates and treated at 3 µM (ChemDiv, Tripos) or 10 µM (Spectrum Collection) compound concentration for 24 h. Mock treatment (DMSO alone) and positive control (nutlin-3) was included in each 96-well plate. Following the incubation, the cells were fixed with paraformaldehyde (PFA) and nuclei were stained with Hoechst 33342 (Molecular Probes).

The cells were imaged using Cellomics ArrayScan 4.5 high-content imaging platform (ThermoScientific) including Zeiss 200 M microscope with a 10x objective (Zeiss) and ORCA-AG CCD camera (Hamamatsu). Target Activation Bioapplication (ThermoScientific) algorithm was used for image acquisition and analysis of the total number of nuclei and percentage of p53DsRed reporter positive cells (responders). A minimum of 1700 cells/well was analyzed. Compounds inducing a minimum of two-fold increase of responders compared to mock were regarded as primary hits. All primary hits were additionally visually inspected for p53 reporter activation and DNA content. In the secondary screen the hits were tested over 0.1–10 µM concentration range. Compounds providing consistent p53DsRed reporter activation were selected for further testing. Z-factor [22] for the screen was 0.632.

### Viability assay

Cells were plated in 96-well plates at a density of 10,000 cells/well. Compounds were added in triplicate and the cells were incubated for three days. Cell viability was assayed using WST-1 cell proliferation reagent (Roche Diagnostics). The experiment was repeated two to three times and results are presented as % viability as compared to the control.

### Human CFU-GM assay

Aliquots of normal bone marrow were obtained from four normal allogenic bone marrow donors granting written informed consent and as approved by the Johns Hopkins Medical Institute Institutional Review Board (J0002). Mononuclear cells were isolated from freshly harvested bone marrow aspirates, seeded at a density of 1×10^6^ cells/ml and incubated with the compounds for 24 hours. The compounds were removed and 5×10^4^ cells were plated and incubated for 14 days [23]. Colonies consisting of greater than 40 cells were counted.

### Immunoblotting analysis

Cellular lysates were prepared and proteins were separated by SDS-PAGE. The following antibodies were used: DO-1 for p53, 2A10 for Hdm2, γH2AX (Upstate), p53Ser15 (Cell Signaling), KAP-1 (BD Transduction Laboratories), KAP-1 Ser824 (Bethyl Laboratories) and anti-p21 (Becton Dickinson). Horseradish peroxidase or biotin-streptavidin-horseradish peroxidase (Dako Cytomation) conjugated secondary antibodies were used. Equal protein loading was verified using Gapdh (p9.B.88, Europa Bioproducts Ltd).

### Immunofluorescence and quantitative image analysis

Cells were grown on glass coverslips and fixed with PFA. γH2AX was detected with anti-H2AX (Ser139) antibody (Upstate) and Alexa488 conjugated secondary antibody (Molecular Probes), nuclei were stained with Hoechst 33342. Images were captured using Axioplan2 fluorescence microscopes (Zeiss) equipped with AxioCam HRc CCD-camera and AxioVision 4.5 software using EC Plan-Neofluar 40x/0.75 objective (Zeiss). p53 immunostaining for cells grown in 96-well plates was performed using DO-1 antibody and Alexa488-conjugate and nuclei were stained with Hoechst 33342. Images were acquired using ArrayScan 4.5 automated imaging platform and analyzed using Target Activation Bioapplication algorithm (Thermo Scientific). The mean average intensity of ≥100 cells was quantified.

### Transcriptional profiling

Cells were treated with the compounds or vehicle DMSO for six hours in four separate experiments and total RNA was isolated. Total RNA (1 µg) was reverse transcribed (Invitrogen SuperScript II reverse transcriptase); double strand cDNA was generated and column-purified (Affymetrix). Biotinylated cRNA was generated through in vitro transcription, fragmented and hybridized to GeneChip human U133A 2.0 arrays for 16 h at 45°C with constant rotation. Hybridized GeneChip was scanned using G3000 GeneArray Scanner (Affymetrix). Image analysis was performed using GeneChip Operating System 1.1.1 (GCOS) software (Affymetrix). Normalized data were imported into Partek Genomics Suite (Partek) software and followed with One-Way ANOVA analysis. Hierarchical clustering analysis was based on Euclidean distance metric and performed using R and Bioconductor software.

### Functional enrichment analysis

Transcripts identified in the microarray were annotated for GO [24] assignments using DAVID database [25]. For enriched GO terms, *P* values from Fisher's exact t-test were cut off at 0.01 on biological process level 4, and GO terms with more than 10 genes with an enrichment score higher than 2.5 were selected. For the KEGG pathways a *P* value <0.05 and an enrichment score greater than 2 in a group of more than 10 genes were used.

### Real-time qPCR

Pooled total RNA (1 µg) was used to generate quantitative real-time PCR (qPCR) standard curve for all genes. Briefly, the pooled RNA was reverse transcribed and used to perform qPCR in triplicate with SYBR GREEN I master mix (Atila Biosystem) on ABI PRISM 7900HT (Applied Biosystems) using primer pairs provided in Methods S1. For quantifying gene expression, qPCR was performed using RNA either from compound or mock treated cells in duplicate in two biological repeats. Transcript quantification was measured by comparison with standard curves as described above. All results were normalized against GAPDH, and coefficient of variation was calculated.

### Flow cytometry

Cell cycle distribution and cell death were assayed with flow cytometry (LSR, Becton Dickinson). Cells were harvested and fixed in 70% ethanol at −20°C followed by RNaseA treatment and staining with propidium iodide. A total of 10,000 counts were collected and data were analyzed using ModFit LT 3.1 software. Cells present in sub-G_1_ population were regarded as non-viable.

### *In vivo* tumor experiment

The animal protocol was approved by the Experimental Animal Committee of Provincial Government of Southern Finland (STH401A), and the animal studies were carried out according to the approved guidelines. An orthotopic model of pleural effusion lymphoma was used to assess the anti-tumor activities of BMH-15 and -22. Female 5–7 weeks old NOD-SCID mice (Charles River) were injected intraperitoneally (i.p.) with 1×10^7^ pleural effusion lymphoma cells carrying a luciferase reporter (BC3luc cells) [26]. For imaging, D-luciferin (100 mg/kg) (Synchem OHG) was injected i.p. followed by imaging 15 min post-injection using Xenogen In Vivo Imaging System (Caliper Life Sciences). Tumor-bearing mice, as verified by bioluminescence, were treated by i.p. injection of the compounds starting on day four post-implantation. The mice received 20 mg/kg compounds BMH-15 and -22 three times a week, while control mice received only DMSO. The mice were imaged prior each treatment. Bioluminescence was quantified and plotted as total flux within given region of interest (ROI) using Igor Pro Carbon analysis software (Caliper Life Sciences). Statistical analysis was carried out using Linear Model ANOVA using R software. *P* values less than 0.05 were regarded as statistically significant.

### Statistical analysis

Shown are means ± SD of at least two independent experiments. Statistical analysis was performed by Fisher's exact *t* test. Data on animal studies was performed using Linear Model ANOVA using R software. Differences were considered statistically significant at *P*<0.05.

## Results

### High-content imaging screen identifies novel p53 activating lead compounds

**Figure 1:**
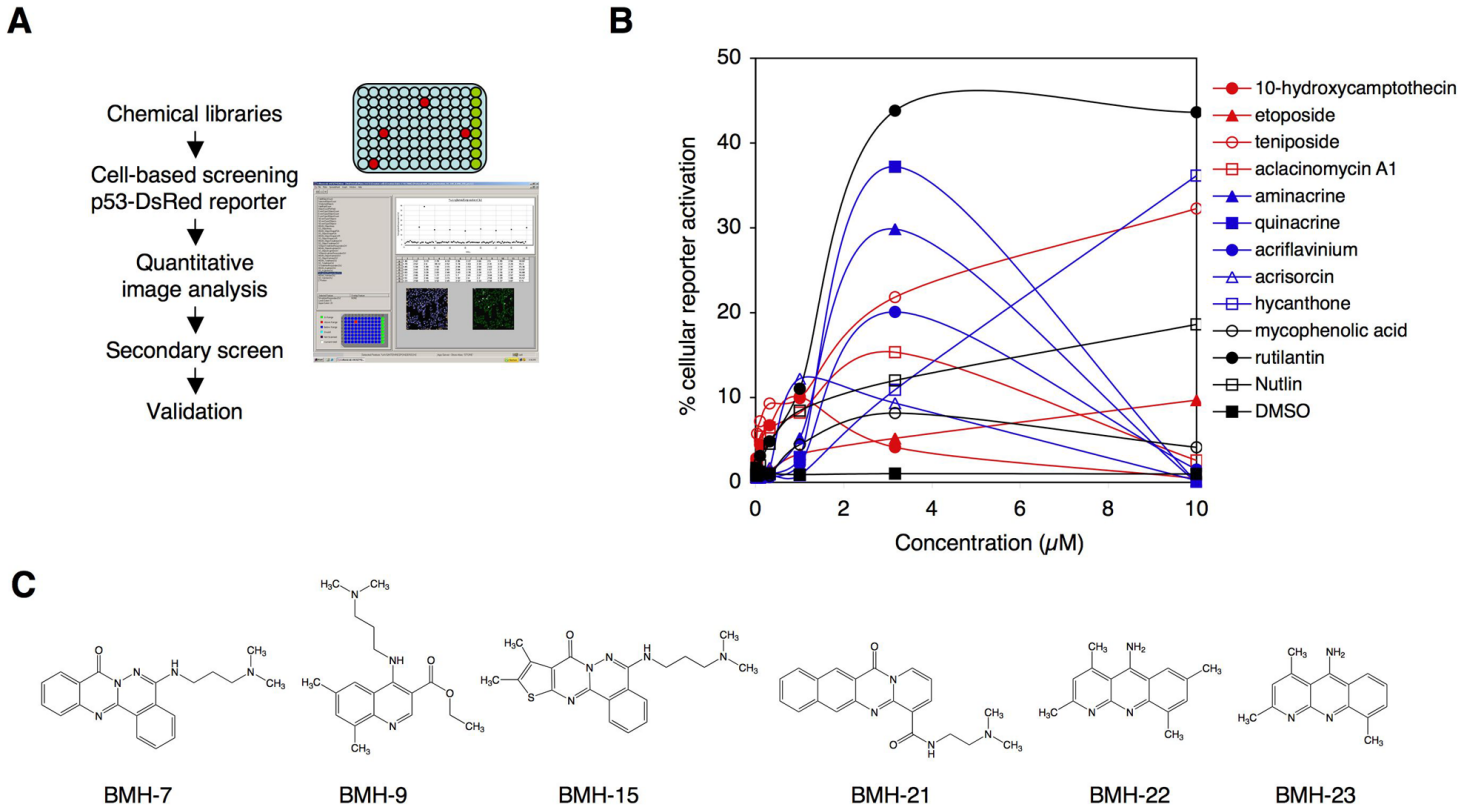
Cell-based high-content imaging screen. A) Screen outline. B) Spectrum Collection experimental drug library was analyzed using the p53 reporter screen. Primary hits were validated at 10-fold concentration range. Color code; red, TOP1/2 inhibitors; blue, DNA intercalating agents; black, other. C) Chemical structures of hit compounds in the large-scale small-molecule compound screen.

An improved protocol for high-throughput cell-based screening was devised. This consisted of generation of a cell line expressing red-emitting fluorescent protein under the control of a p53 consensus promoter (p53DsRed) [21]. In contrast to the previously published screens using a similar reporter element, we adapted the screen to a strictly cell-based analysis using a fully automated high-content imaging and quantitative image analysis platform. This facilitated scoring of signal intensities in individual cells allowing detection of the reporter activation under conditions where p53 activation leads to a significant reduction in cell viability (Figure 1A).

We first validated the p53 high-content imaging assay and screened a library consisting of 2000 therapeutic and experimental drugs. We identified and further validated eleven drugs that activated and stabilized p53, of which four are for the first time described here (Figure 1B and Table S1). Of the eleven, five drugs intercalate with DNA, and four are topoisomerase 1 (TOP1) or topoisomerase 2 (TOP2) inhibitors. The results indicated that the p53 high-content imaging screen can be applied for testing of both novel and known drugs for their anti-tumor activities.

We then proceeded to screen synthetic small-molecule chemical compound libraries (40,000 compounds) at a relatively low compound concentration (3 µM) in order to increase the likelihood to select for potent p53 activating molecules. Nine small-molecule compounds that consistently activated the p53 reporter at low micromolar concentrations were discovered. The chemical structures of six compounds amenable for larger scale syntheses are presented in Figure 1C and were used in subsequent studies. Searches for previously reported p53 activating small-molecule compounds indicated that the lead compounds identified here, BMH-7, BMH-9, BMH-15, BMH-21, BMH-22, and BMH-23 were novel. Furthermore, among the identified leads, there were two sets of compounds (BMH-7 and BMH-15, BMH-22 and BMH-23) that were structurally highly related to each other (Figure 1C). The compound chemical characteristics indicated excellent predictions for the compound solubility, permeability and oral bioavailability (Lipinsky's rule of five) [27] (Table S2).

**Figure 2:**
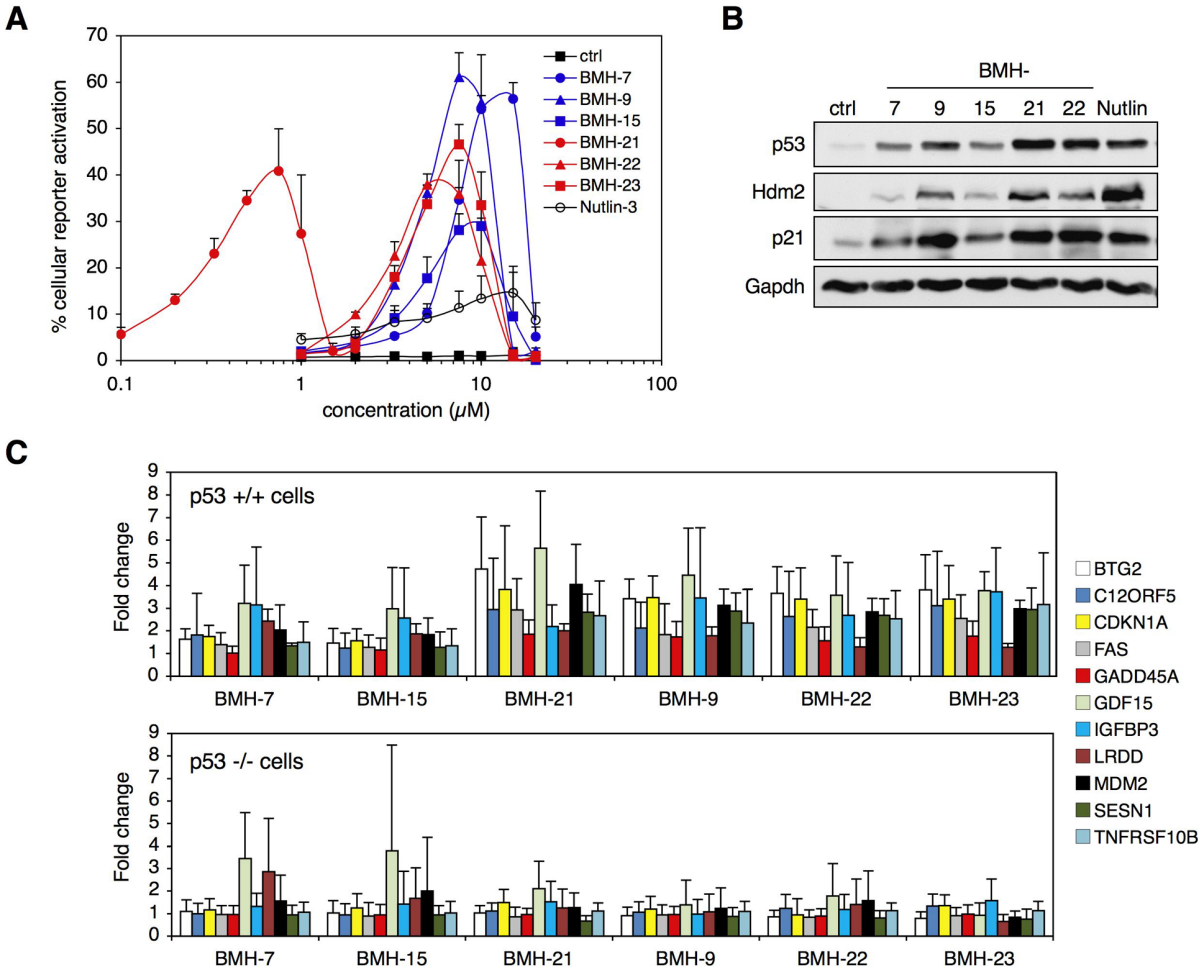
Lead compounds activate p53 pathway. A) p53DsRed reporter activation. A375 cells stably expressing p53DsRed reporter were incubated with the lead compounds and nutlin-3 for 24 hours, followed by high-content image analysis. The percentage of cells expressing p53DsRed reporter is shown. The experiment was performed in triplicate and represents ≥1700 cells per datapoint. Error bars represent SD. B) p53 stabilization and target protein regulation. A375 cells were treated with BMH-7, -9, -15, -22, nutlin-3 (5 µM), and BMH-21 (0.5 µM) for 24 hours followed by analyses for p53, Hdm2 and p21 protein levels. Gapdh was used as a loading control. C) p53-dependent gene regulation. Isogenic HCT116 p53+/+ and p53−/− cells were treated with the compounds followed by qPCR for p53 target genes. Fold induction of the relative levels of the transcripts in the p53+/+ and p53−/− cells are shown. Data represent duplicate biological experiments and duplicate qPCR reactions. Error bars represent SD.

We then assessed the ability of the compounds to activate the p53 pathway. Dose-titration experiments of the leads indicated that all were highly potent inducers of the p53DsRed activation (30–65% positive cells) as compared to nutlin-3 (15% positive cells) and were active at nanomolar (BMH-21) to low micromolar range (BMH-7, BMH-9, BMH-15, BMH-22, BMH-23) (Figure 2A). Notably, high compound concentrations (≥10 µM) caused extensive cell death eventually quenching the reporter signal. All compounds markedly stabilized p53 and increased the levels of its transcriptional targets, Hdm2 and p21Cip1 (Figure 2B), whereas p53 mRNA levels were in fact suppressed by four out of six compounds (Figure S1).

To further assess the regulation of known p53 target genes by the lead compounds we undertook qPCR analysis of eleven known p53 targets. In order to validate p53 dependency, a comparison between the responses in isogenic HCT116 p53+/+ and p53−/− cell lines [28] was conducted. The induction of the target genes by BMH-9, -21, -22, and -23 was clearly dependent on p53, whereas BMH-7 and BMH-15 had more restricted capacity to p53 dependent gene regulation (Figure 2C). These analyses show extensive activation of p53 downstream signaling by four lead compounds, and more limited by two.

### Novel lead compounds are antitumorigenic

**Figure 3:**
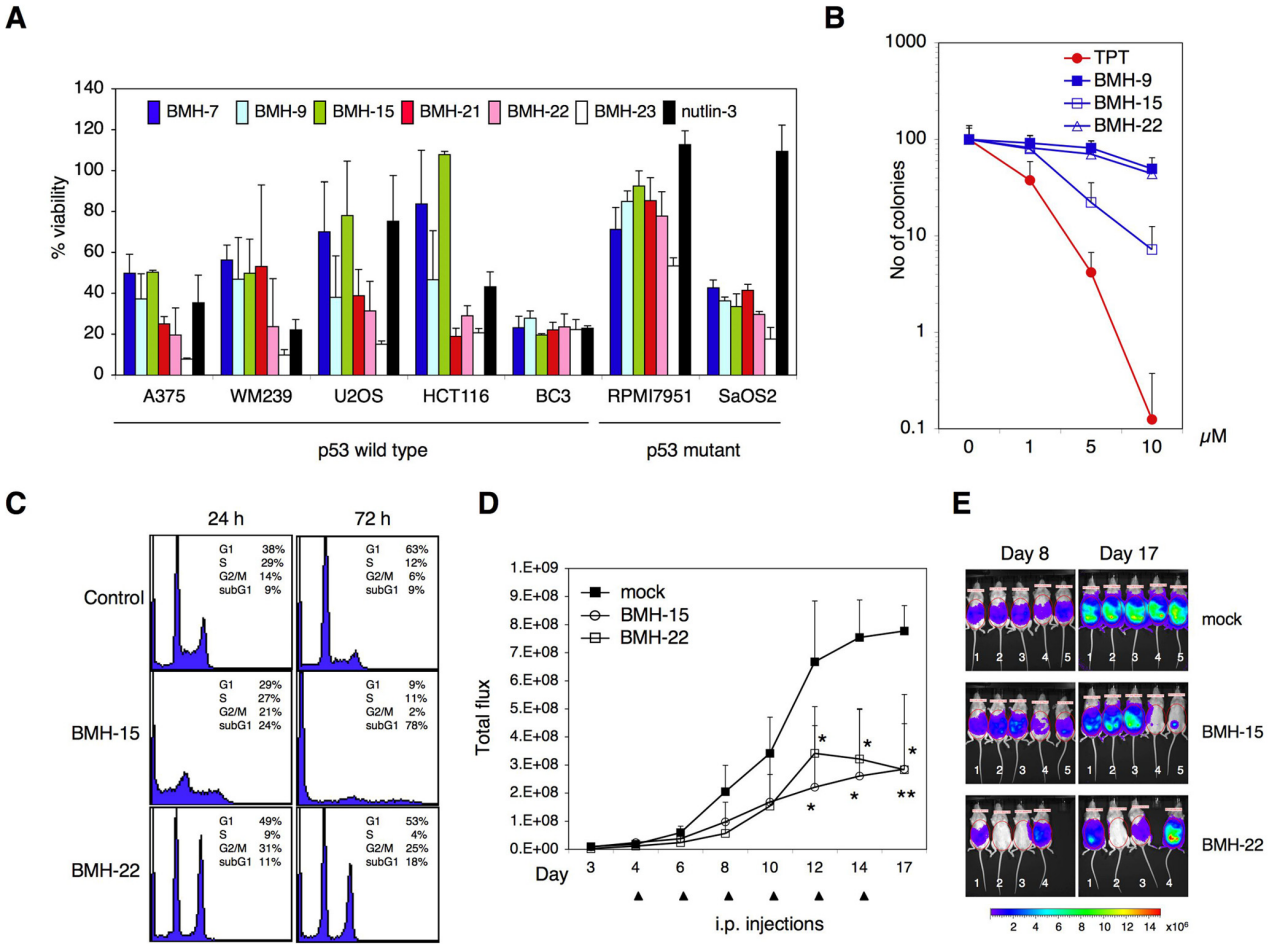
Lead compounds are anti-tumorigenic. A) Tumor cell lines (A375, WM239, RPMI7951 melanoma, U2OS and SaOS2 osteosarcoma, BC3 lymphoma, and HCT116 colon adenocarcinoma cells) were incubated with BMH-7, -9, -15, -22, -23, nutlin-3 (10 µM), and BMH-21 (1 µM) in triplicate for three days. Cell viability was determined using WST-1 cell proliferation assay. Cell viability in control is set as 100%. Error bars represent SD. B) Dose-response curves of normal human CFU-GM. Shown is the clonogenic recovery of normal CFU-GM derived from four healthy individuals, treated with topotecan (TPT), BMH-9, BMH-15 and BMH-22. Error bars represent SD. C) BC3 cells were treated with BMH-15 and BMH-22 (10 µM) for 24 and 72 hours followed by flow cytometry. Cell cycle distribution and fraction of sub-G_1_ cells are indicated. D) NOD-SCID mice were injected i.p. with BC3luc cells, and tumors were allowed to establish for three days. Tumor take was confirmed by imaging and the mice were treated with i.p. injection of BMH-15 and BMH-22 (20 mg/kg) three times a week. Control animals received DMSO. The mice were imaged prior to each treatment and bioluminescence was quantified and plotted as total flux within a constant ROI. BMH-15 and BMH-22 caused significant reductions in tumor signals (ANOVA analyses, BMH-15, days 12, 14, 17, *P* values 0.0281, 0.0138, 0.0131, n = 5, respectively; BMH-22, days 12, 14, 17, *P* values 0.0259, 0.0160, 0.0045, n = 4, respectively; mock, n = 5). Error bars, SD. E) Bioluminescence images of the mice at day 8 and day 17. Heatbar indicates the bioluminescence intensities.

Given the prominent activation of the p53 responses, we analyzed the effects of the compounds on the viability of several tumor and normal cell lines. The lead compounds potently suppressed the viability of various tumor cells, albeit to different degrees (Figure 3A). These included cell lines wild-type, mutant and null for p53, and showed that the antiproliferative activity of only BMH-9 was somewhat dependent on p53 (Figure S2). Comparison of the lead compound effects in three normal human cell lines and tumor cells indicated higher resistances of the normal cells to the compound antiproliferative activity (Table S3). We also assessed the potential toxicity of three representative compounds against human hematopoietic progenitors. As shown in Figure 3B, BMH-9 and BMH-22 exhibited essentially no cytotoxicity and BMH-15 produced only a 10% inhibition of granulocyte-macrophage colony-forming units (CFU-GM). In contrast, topotecan, a TOP1 poison and known hematopoietic suppressor, had prominent toxicity. The results indicate that the lead compounds are potential novel anticancer agents without excessive toxicity in several human primary cells.

To validate the potential use of the compounds *in vivo*, we first utilized an *ex vivo* tissue culture model of human prostate tissues [29, Methods S1]. The lead compounds BMH-7, BMH-9, BMH-15, BMH-21 and BMH-22 clearly induced p53 expression in the epithelial compartment of human prostate tissues indicating tissue permeability (Figure S3). To assess for potential anti-tumor activity *in vivo* in a mouse model, we used BC3 pleural effusion lymphoma cells that we have previously shown to be highly sensitive to p53 pathway activation [20]. Two compounds (BMH-15, BMH-22) available in larger scale required for the animal studies were tested. Initial flow cytometric analysis of the compound effects in BC3 cells indicated rapid and extensive cell death (BMH-15), or pronounced G_2_/M phase arrest and cell death (BMH-22) (Figure 3C). We then employed an orthotopic model of B cell lymphoma of BC3luc cells expressing luciferase-reporter [26]. Following BC3luc cell tumor establishment, the mice were treated by i.p. injection (20 mg/kg) three times weekly at which times the tumor bioluminescence was recorded (Figure 3D and 3E). At the given doses, BMH-15 and BMH-22 produced significant (*P*≤0.013) anti-tumor activity. None of the compounds showed any toxic effects in mice as determined by weight curves, well-being, or histology of organs following administration at a similar dose regime for over a three-week period (Figure S4, Methods S1). We conclude that the compounds display significant anti-tumor activity without overt toxicity.

### The lead molecules act on DNA but diverge in their potential to cause DNA damage

Based on the planar heteroaromatic ring structures we addressed the DNA intercalation properties of the compounds. DNA intercalation was assessed by changes in the compound absorbance spectrum by UV-VIS in the presence of DNA. All compounds displayed hypochromic and/or bathochromic shifts befitting DNA binding [30] (Table S4, Methods S1).

DNA intercalators lead to lengthening of the double helix and unwinding. We therefore used a DNA unwinding assay, which also distinguishes between a DNA intercalator and a putative TOP1 inhibitor [30], [31]. Concordant with the UV-VIS assays, all compounds were found to cause extensive DNA unwinding, but not TOP1 inhibition (Figure S5, Methods S1 and data not shown). Based on these assays, all lead molecules intercalate with DNA.

**Figure 4:**
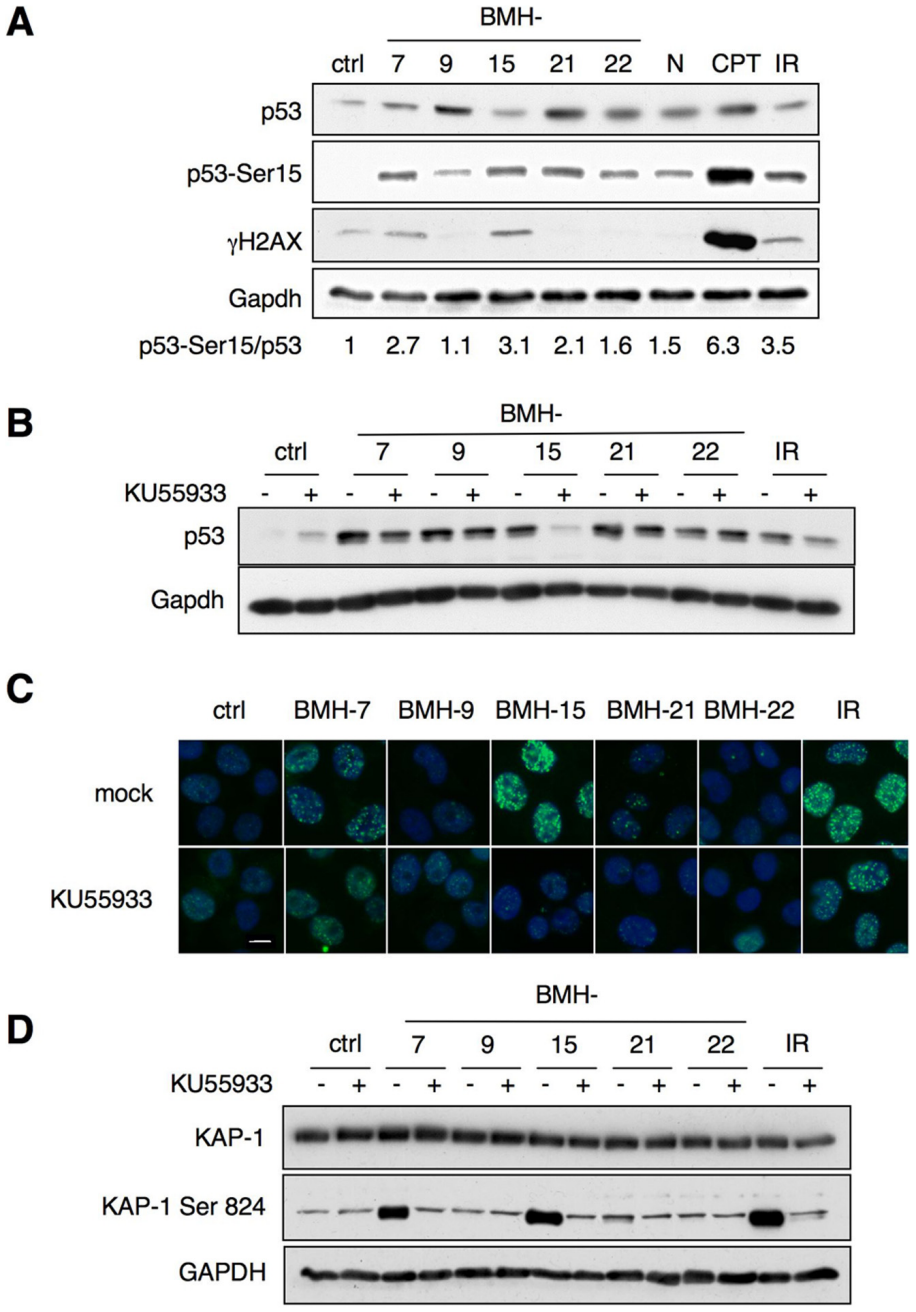
Lead compounds have both DNA damage response-dependent and independent activities. A) A375 cells were treated with compounds BMH-7, -9, -15, -22 (5 µM), and BMH-21 (0.5 µM) for 8 hours and cell lysates were analyzed by immunoblotting for p53, p53Ser15, and γH2AX. The mean fold induction of p53Ser15, as compared to total p53, is shown below the blots (n = 2). All samples were normalized to Gapdh. B) Cells were treated with ATM inhibitor KU55933 (10 µM) for 90 min, followed by addition of BMH-7, -9, -15, -22 (5 µM), BMH-21 (0.5 µM), or ionizing radiation (IR) (10 Gy), and incubation for 6 hours. p53 stabilization was analyzed by immunoblotting. C) γH2AX foci formation of cells treated as in Figure 4B was analyzed by immunofluorescence. Nuclei were stained with Hoechst 33342. Scale bar, 10 µm. D) Cells were pretreated with KU55933 as in Figure 4B, followed by addition of the compounds for 30 min. KAP-1 and KAP-1 Ser824 was analyzed by immunoblotting.

As p53 is highly sensitive to DNA damage [2], [32] and as DNA intercalators may cause DNA damage, we assessed whether the compounds cause activation of the DNA damage signaling pathways. The analysis of compound-treated cells for ATM target proteins γH2AX, Ser15 phosphorylated p53 and Ser824 phosphorylated KAP-1 revealed that DNA damage response was activated by BMH-7 and BMH-15 (Figure 4A). To further estimate the dependency of p53 induction on activated ATM-cascade, we treated the cells with the compounds in the presence or absence of a specific ATM inhibitor KU55933 [33]. p53 stabilization, γH2AX foci formation and KAP-1 phosphorylation by BMH-7 and BMH-15 were ATM-dependent (Figure 4B and 4C and 4D). BMH-15 induced a prominent γH2AX response also in human prostate tissue (Figure S3B). We conclude that while BMH-7 and BMH-15 elicit an ATM-dependent DNA damage response, more importantly, BMH-9, BMH-21, and BMH-22 do not. BMH-23, analyzed separately, did also not activate ATM-pathway (data not shown).

### Transcriptional profiling reveals highly similar cellular responses

**Figure 5:**
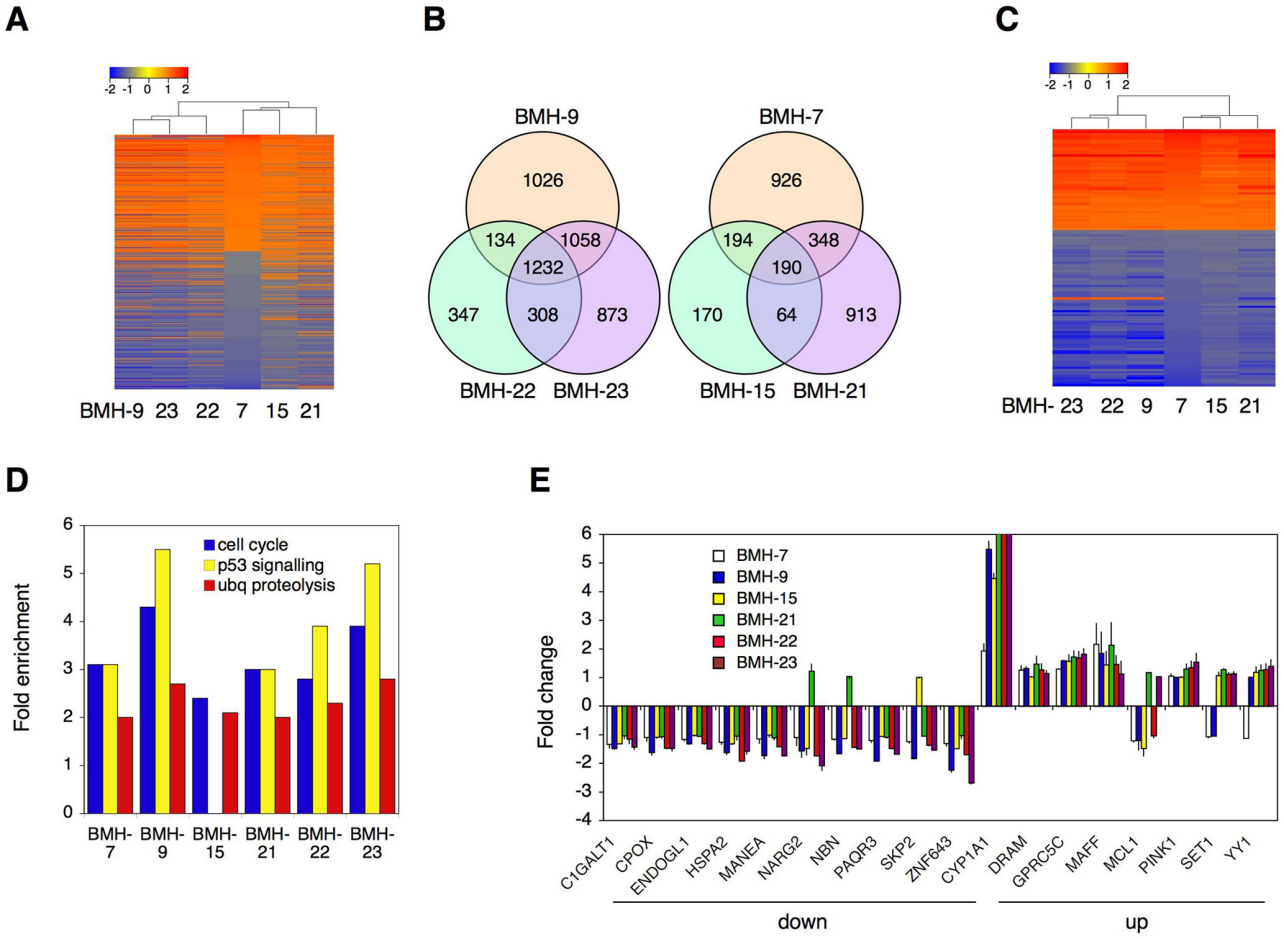
Transcriptional profiling and gene expression analysis of the lead compounds. A) MCF-7 cells were treated with BMH-7, -9 (10 µM), BMH-15, -22, -23 (5 µM), and BMH-21 (0.5 µM) for 6 hours, and control cultures were mock-treated with DMSO. Total RNA was isolated from four independent experiments. Differentially expressed transcripts (*P*<0.01, total of 5926 transcripts) were identified by One-Way ANOVA analysis and hierarchical clustering was performed using R software. Dendrograms and heat map values are shown. B) Venn diagrams of transcripts shared by BMH-9/22/23 and BMH-7/15/21. C) Hierarchical clustering of 118 target genes shared by all compounds. D) KEGG pathways. Pathways extensively shared are shown (*P*<0.05, enrichment score >2.0, over 10 targets/category). E) qPCR verification of transcripts identified by profiling. qPCR was performed on randomly selected eight up-regulated and ten down-regulated genes. Fold change as compared to GAPDH is shown. Note that CYP1A1 bars for BMH-21, -22, -23 are truncated (actual values 55.2, 10.1, and 6.2, respectively). Data represent duplicate biological experiments and duplicate qPCR reactions. Error bars represent SD.

p53, when activated, exerts an extensive transcriptional program affecting hundreds of target genes [34], [35]. To validate the extent to which the lead compounds activate p53 pathway and to gain further information of potential other pathways affected, we used transcriptional profiling. Analysis of the gene expression patterns showed a total of 5926 transcripts undergoing significant up – or down regulation (GSE #12666, Dataset S1). Hierarchical clustering indicated similar transcriptional profiles despite the chemical diversity of the compounds. BMH-9 was closely clustered with BMH-22 and BMH-23 and BMH-21 with BMH-7 and BMH-15 (Figure 5A). Further analysis, as shown by Venn diagrams, indicated extensive mutual sharing of transcriptional targets within the clusters (Figure 5B). All compounds shared a total of 118 targets with a high degree of similarity of the elicited responses (Figure 5C and Dataset S2). To provide indications for the cellular programs activated by the compounds we performed GO assignments of the transcripts using DAVID analysis platform [24], [25]. The analysis indicated a significant enrichment of DNA damage checkpoint, response and repair genes, and cell cycle genes (see Table S5 for complete analysis). We further queried KEGG for specific pathways affected. The analysis showed marked enrichment of pathways affecting cell cycle, p53 signaling and ubiquitin mediated proteolysis (Figure 5D and Table S6). To verify the results of the transcriptional profiling, we performed qPCR of a number of transcripts shared by the compounds (8 up- and 10 down regulated transcripts). QPCR analysis closely correlated with the transcriptional profiling (Figure 5E).

### Genomic signatures of the lead compounds reveal similarities to known drugs

**Figure 6:**
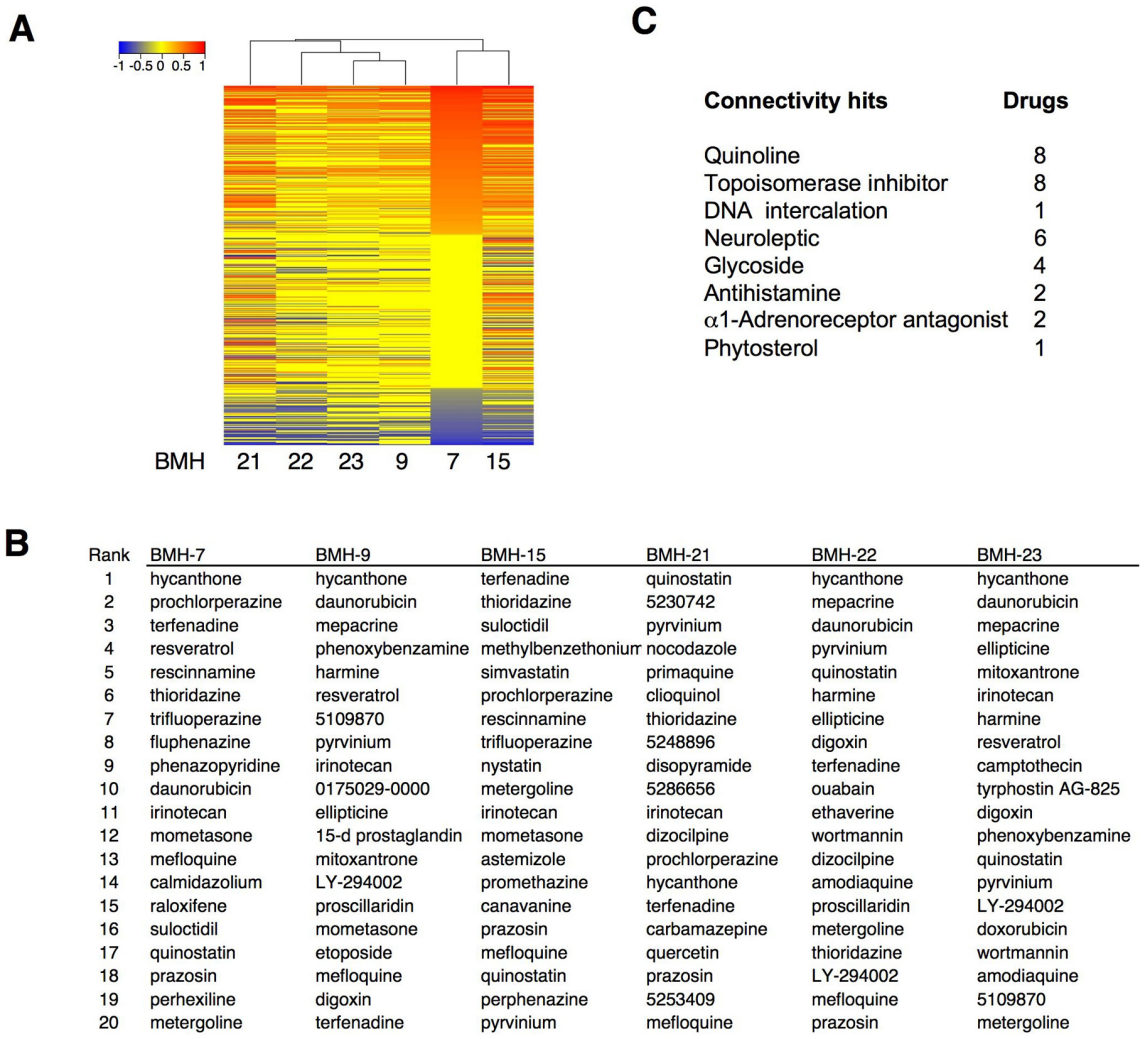
Connectivity map analysis identifies drugs with similar cellular responses. A) Hierarchical clustering of connectivity scores. Gene expression signatures (1000 genes from each compound profile) were queried against Connectivity Map Database 2.0. Dendrogram of the ranking order expression signatures is presented. B) Top-ranking connectivity signatures. C) Classification of top-ranking connectivities.

To evaluate whether the lead compounds share similarities between drugs with known mechanisms of action, we compared the gene expression profiles elicited by the compounds to those at the Connectivity Map database [36]. The Connectivity Map comprises gene expression profiles of over 2000 experimental and known drugs. Comparison of the query transcriptional profile provides a ranked order of agents triggering similar responses. Clustering analysis of profiles ranking with the lead compounds indicated a high degree of similarity both in terms of positively and negatively ranking hits (Figure 6A). High-ranking scores were obtained for eight topoisomerase inhibitors, eight quinoline derivatives, many of which are used as anti-helminitic drugs, and hycanthone, which was the top-scoring hit with four compounds (Figure 6B and 6C). These findings are fully consistent with the identified high-content screen hits in the Spectrum Collection (Figure 1B). Among the highest scoring hits in the connectivity analysis, 30.5% have previously been implicated in p53 pathway activation (Table S7). Interestingly, the analysis identified frequent hits of drugs in clinical uses other than cancer. These included six phenothiazines, four glycosides, antihistamines and a1-adrenoreceptor antagonists (Figure 6B). However, none of these drugs tested separately affected p53 or caused activation of DNA damage signaling (not shown), suggesting that they may share other parallel activities, which may include anti-tumor properties.

## Discussion

We present here the discovery of novel small-molecule lead compounds and detailed studies of their anti-tumor activities and mechanisms of action. The lead compounds caused death of colon, melanoma, osteosarcoma, and lymphoma tumor cells at sub-low micromolar concentrations while maintaining viability of normal primary cells and human bone marrow. The compounds showed tissue permeability in a human *ex vivo* prostate tissue model as evidenced by p53 induction, supporting their use *in vivo*, and two compounds effectively reduced B cell lymphomagenesis in an orthotopic mouse model. Five of the compounds displayed striking effects by decreasing cell viability independently of p53. This underscores that the compounds in fact trigger upstream p53 activating events and activation of potent cytotoxic pathways.

p53 is highly sensitive to DNA damage stress [2], [32]. However, only two of the compounds (BMH-7 and BMH-15) activated ATM-dependent DNA damage signaling, i.e. acted as potentially genotoxic agents. The absence of the DNA damage response by four lead compounds indicates that their actions are unrelated to those directly causing DNA damage. The absence of DNA damage response may in fact be a preferable mechanism of action by potentially lowering normal cell toxicity, including genotoxicity. Notably, BMH-21, which had the most potent antitumorigenic activity, did not activate DNA damage signaling. Importantly, the improved screen protocol used here not only allowed selection of cell permeable compounds, but also facilitated the identification of lead molecules with antitumor activities in the nanomolar range.

All compounds were found to intercalate with DNA. DNA is an extremely attractive target for cancer therapeutics. It poses exquisite sequence-specificity and unique structures at different metabolic states, and is also a widely targeted molecule by many anticancer drugs [37], [38]. These interact with DNA typically through three modalities, namely DNA intercalation, groove binding and covalent interactions causing cytotoxicity and therapeutic advantage [30], [38]–[42]. DNA intercalators, beyond those that induce DNA damage, lead to local structural changes in DNA, including unwinding and lengthening of the DNA helix [30], [37], [38]. These events may lead to alterations in DNA metabolism, and halter transcription and replication, and induce p53 [43]. Interestingly however, the lead compounds cause distinct transcriptional programming, which includes both transcriptional induction and repression, and are consistent with activation of pathways regulating cell cycle progression, p53 pathway, ubiquitin-dependent proteolysis and DNA damage surveillance (Figure 5). These findings indicate that the compounds do not cause a general transcriptional repression, but rather, selective regulation. Furthermore, this transcriptional programming seems to be largely shared by the lead compounds, especially those by BMH-9, BMH-22 and BMH-23. The implication of these findings is that the compounds may also selectively alter DNA metabolism in a manner that may be dependent on the DNA sequence, conformation or interactions of the compounds with chromatin proteins.

The high frequency of quinoline-derivatives among the lead compound profiles and the chemical screens, and their known property of DNA intercalation [39], raises the possibility that this property may predispose to p53 induction or antitumor activity. Many of the agents identified by the chemical screens or lead profiling analyses have recognized toxicities and some, carcinogenic effects. Therefore, activation of p53 may reflect severe cellular stress that may culminate in the elimination of tumor cells or, in susceptible normal cells, toxicity. However, the novel lead compounds had low to non-existent human bone marrow toxicity and no *in vivo* toxicity in mouse. Considering their favorable pharmacokinetic predictors and demonstrated antitumor activity we propose that they represent promising novel anticancer lead compounds.

The lead compounds identified in the chemical screen were structurally diverse, although were represented by two closely related pairs. Though all contained polycyclic heteroaromatic rings, these varied from two –to four-ringed structures. Based on the structural characteristics, it would have been difficult to ascribe that they possess similar biological activities. However, cellular assays and transcriptional profiling showed that the lead compounds displayed strikingly similar properties. Connectivity map analysis showed that the lead molecules shared transcriptional signatures with DNA intercalators and topoisomerase poisons. However, detailed analyses for potential topoisomerase inhibitory activities of the compounds were negative (not shown). Furthermore, the genomic signatures triggered by the compounds suggested unexpected biological parallels with neuroleptics and other widely used drugs. Due to the wide, and often long-term use of these drugs, their activity in non-target tissues could present as undesirable side effects or as an unexpected health benefit. Interestingly, a large population-based cohort study of the use of phenothiazines has indicated a decrease in risk of rectal, colon and prostate cancer [44]. Prazosin, and other quinazoline-based adrenoreceptor antagonists doxazosin and terazosin, are used as hypertensive drugs and for prostate hyperplasia, and recently, suggested to possess antitumor properties [45], and to decrease risk of prostate cancer in men [46]. Cardiac glycosides digoxin, ouabain, quercetin and proscillaridin, all providing high connectivity scores, have been associated with topoisomerase inhibition, cellular DNA damage response, cytotoxicity of breast cancer cells and increased breast cancer survival [47], [48]. Strikingly, we note that all of the recently discovered hypoxia-inducible factor-1 inhibitors (daunorubicin, acriflavin, digoxin, ouabain and proscillaridin A) are among the hits identified here [49]. These findings underscore that the lead molecules share remarkable parallels with several pharmaceutics amenable towards repurposing as cancer therapeutics.

In conclusion, this study represents an extensive analysis of drug-based mechanisms activating the p53 pathway, emphasizes the dominant role of DNA intercalation in p53 pathway activation and identifies novel regulators of DNA topology with promising anticancer properties.

## Supporting Information

Methods S1 Supplementary methods.
(0.16 MB PDF)

Dataset S1 Significant transcriptional changes caused by the compounds (5926 transcripts, P<0.01).
(3.03 MB XLS)

Dataset S2 Genes regulated by all compounds (118 genes, P<0.01).
(0.08 MB XLS)

Figure S1 qPCR analysis of TP53 mRNA expression. MCF-7 cells were incubated with BMH-7, -9 (10 µM), BMH-15, -22, -23 (5 µM), and BMH-21 (0.5 µM) for 6 h, and control cultures were mock-treated with DMSO (n = 4). Total RNA was isolated and qPCR performed for TP53. The values were normalized according to GAPDH. Error bars represent SD.
(0.15 MB PDF)

Figure S2 p53 dependency of cell viability. Lead compound effect on p53 isogenic HCT116 cells. HCT116 p53+/+ and p53−/− cells were cultured in the presence of BMH-7, -9, -15, -22, -23 (5 µM), BMH-21 (0.5 µM), and nutlin-3 (5 µM) for 72 h followed by counting of the cells. The relative viability response as adjusted to controls in the p53+/+ as compared to p53−/− cells is shown. Error bars represent SE.
(0.16 MB PDF)

Figure S3 Human *ex vivo* prostate tissue. Fresh prostate tissues were obtained from radical prostatectomies, and the sections were incubated with BMH-7 (20 µM), BMH-9 (20 µM), BMH-15 (20 µM), BMH-21 (2 µM), and BMH-22 (20 µM) for 24 h, fixed and stained for p53 and DNA, and in (B) also for gamma-H2AX. Images were captured using confocal microscopy (A) or wide-field microscopy (B). Prostate glands are indicated by white dashed lines. Scale bar, 50 µm.
(0.53 MB PDF)

Figure S4 *In vivo* toxicity. Mice were injected intraperitoneally with BMH-7, -9, -15, -22 (20 mg/kg), and BMH-21 (2 mg/kg) three times a week for three weeks in 30 µl DMSO. Control animals received only the DMSO vehicle. The mice were sacrificed and organs (thymus, spleen, intestine, liver and kidney) were collected for histological examination. No acute or chronic toxicities were observed based on the histological hematoxylin-eosin analyses. Similarly, there were no changes in the weight curves of mice undergoing the above treatment regimen (N = 2 for each treatment group) (data not shown).
(1.16 MB PDF)

Figure S5 DNA unwinding. TOP1 (2 U) was added to plasmid DNA to allow full relaxation of the plasmid (Rel). Subsequently, an excess of TOP1 (20 U) and increasing amounts of compounds (BMH-7, -9, -15, -22, -23, 0.01-5 µM; BMH-21, 0.001-0.5 µM) were added and incubated for further 1 h at 37°C. The reaction was quenched and the samples were analyzed by agarose gel electrophoresis. Note appearance of DNA topomers (Rn) and supercoiled DNA (Sc) due to intercalation. D, DMSO control, EB ethidium bromide.
(0.22 MB PDF)

Table S1 p53 activating drugs in the Spectrum Collection.
(0.06 MB PDF)

Table S2 Lipinsky rule of five.
(0.06 MB PDF)

Table S3 *In vitro* normal and melanoma cell line viability responses.
(0.07 MB PDF)

Table S4 DNA intercalation of the compounds by UV-VIS.
(0.06 MB PDF)

Table S5 GO categories of transcriptional targets.
(0.06 MB PDF)

Table S6 KEGG pathways of transcriptional targets.
(0.07 MB PDF)

Table S7 Summary of top-ranking connectivities.
(0.06 MB PDF)
