# Supplementary material for: Identification of Novel p53 Pathway Activating Small-Molecule Compounds Reveals Unexpected Similarities with Known Therapeutic Agents
Source: PLoS One. 2010 Sep 27;5(9):e12996. doi: 10.1371/journal.pone.0012996 (PMC2946317; doi:10.1371/journal.pone.0012996)
Supplement: Methods S1 — Supplementary methods. (0.16 MB PDF) [file pone.0012996.s001.pdf]

Supplementary Methods

DNA Unwinding Assay. DNA unwinding reaction (1) was carried out in an assay buffer consisting of 50 mM Tris-HCl (pH 7.5), 20 mM KCl, 1 mM EDTA, 1 mM dithiothreitol, and 300 µg/mL BSA. Relaxed pUC19 plasmid DNA substrate was prepared by incubating topoisomerase 1 (TOP1) (2 U) with supercoiled plasmid (100 ng) in the assay buffer for 2 h at 37˚C. Subsequently, an excess of TOP1 (20 U) and increasing amounts of compounds in DMSO (BMH-7, -9, -15, -22, -23, 0.01-5 µM; BMH-21, 0.001-0.5 µM) were added and incubated for 1 h at 37ºC. The final concentration of DMSO in all reaction mixtures was kept to 0.5%. The reaction was quenched by adding SDS (1%) and proteinase K (100 µg/mL), and incubated for 1 h at 45˚C. Samples were loaded into an 1% agarose gel containing 0.1% SDS, run in TBE/0.1% SDS buffer, followed by staining with ethidium bromide and visualization under UV illumination using a Bio-Rad ChemiDoc XRS apparatus.

DNA Binding by UV-VIS. UV-VIS analyses are as modified from references (2) and (3).

Concentration of calf thymus DNA was determined by absorbance measurements at 260 nm using ε = 12,824 M $(bp)^{-1}$ cm^{-1}. Compounds were dissolved in aqueous buffers and the absorption spectrum of the compound in the absence of DNA was recorded over a wavelength range of 200-750 nm at 25˚C using SpectraMax M5 (Molecular Devices) spectrophotometer. Absorbances after sequential additions of the DNA were recorded until no further decrease in absorption was observed. This value was recorded as the absorbance, A_b, of the totally bound compound. The extent of DNA binding/intercalation was measured by determining the shift (bathochromic shift) in λ_{max} between A_f and A_b [i.e., λ_b-λ_f] and the percent decrease in the absorbance (hypochromic shift) of bound compound [i.e., 100 x $(A_f – A_b)/A_f$] from the λ_{max} values.

Proliferation Assays. For cell proliferation assays isogenic HCT116 cells (p53+/+ and p53-/-) were plated in 6 well plates at a density of 50,000 cells/well in McCoy's 5A medium with 10% FBS and penicillin-streptomycin (100 U/ml and 100 µg/ml, respectively) and were grown at 37°C supplemented with 5% CO_2. Compounds diluted in DMSO were added and the cells were incubated for 24 and 72 h. Cells were harvested, loaded onto a cell counting chamber and counted using Cellometer™ Auto T4 (Nexcelcom, Bioscience) cell counter.

Ex Vivo Human Tissue Culture and Confocal Imaging. The protocol for ex vivo prostate tissue cultures has been described previously in detail (4). Tissues were

obtained with written informed consent and approval (Ethics Committee, Department of Surgery, Hospital District of Helsinki and Uusimaa, permit number 390/E6/06). Tissues representing histologically normal areas were bored from radical prostatectomy specimens, and sliced at 0.5 mm with a Krumdieck precision tissue slicer (Alabama Research and Development Corporation). The tissue slices were incubated with the indicated compounds or mock-treated (DMSO), fixed, embedded in paraffin and cut at 4 μm. The tissues were then deparaffinized, rehydrated, and the antigens retrieved in Tris-EDTA buffer (pH 9.0) using a microwave oven. The slides were blocked in 5% milk/0.25% Triton X-100 in TBS with 2% goat nonimmune serum (DakoCytomation) for 30 minutes. p53 was detected with DO-7 (DakoCytomation) and Alexa 488-conjugated secondary antibody (Molecular Probes). Nuclei were counterstained with Hoechst 33342. Confocal images were acquired with LSM 5 Duo confocal laser scanning microscope (Zeiss) equipped with Plan-Apochromat 40x/1.3 oil objective. Images were captured with scan zoom 1 and line scan averaging of 4 using LSM AIM software Rel. 4.2 with Physiology Module.

In vivo toxicity. The animal protocol was approved by the Provincial Government of Southern Finland (STH561A). Acute and chronic toxicity of the compounds was tested in FVB/N mice. Female 5-7 weeks old (Harlan; two animals/compound dose) were injected with the compounds (BMH-7, -9, -15, -22 [20 mg/kg] and BHM-21 [2 mg/kg] in 30 μl DMSO) i.p. three times a week for three weeks. Two animals received vehicle only. The animals were weighed prior to injections and monitored for any signs of toxicity, distress or unusual behavior. The animals were sacrificed three days after receiving the final injection. Liver, kidney, spleen, small intestine and thymus were excised and fixed in 10% formalin overnight, and mounted in paraffin. Hematoxylin-eosin staining (HE staining) of the tissues was performed using standard immunohistological techniques.

qPCR primers

Oligo symbol	Tm	Sequence	Product length	Gene symbol	Primer positions (nt)
CYP1A1-F	59.69	cccttattgctgtcctggag	87	CYP1A1	2068 - 2483
CYP1A1-R	59.95	agtcctggtgcctggatatg			
DRAM-F	60.42	agcccatgtagacagcttcg	111	DRAM	2365 - 2793
DRAM-R	59.48	agaggtacctgccactgagc			
GPRC5C-F	59.98	cagagcatgttcgtggagaa	86	GPRC5C	1257 - 1761
GPRC5C-R	59.99	ttgtacccgctgtatggtga			
MAFF-F	59.97	ggtgacacggatggaagagt	123	MAFF	1561 - 2071
MAFF-R	59.86	ctatttgccacagcagtcca			
MCL1-F	59.87	gcagtgagggcttaggacac	114	MCL1	3521-4020
MCL1-R	59.76	cccgaactacgtagccagtc			
PINK1-F	59.96	tttgtgggcaggtatcaaca	104	PINK1	2180-2680
PINK1-R	59.87	ctgcatgttgacgctgattt			
SET1-F	58.79	aactgtccctgctttctggt	114	SET	2436-2936
SET1-R	59.98	gcattagggtgcttctctgc			
YY1-F	60.99	ctgccagatgctgatgttca	125	YY1	2092-2592
YY1-R	58.62	ttgccctttctgttacatgg			
C1GALT1-F	59.2	tgaagctttaaatgagctgtgaa	113	C1GALT1	1231 - 1741
C1GALT1-R	59.93	cctctacctggccaccatt			
CPOX-F	59.73	tgttgggattctctgcacttt	99	CPOX	1745 - 2268
CPOX-R	57.93	tctcaacttccacacagagatg			
ENDOGL1-F	60.2	gaattccaagtgagcctcca	118	ENDOGL1	672 - 1172
ENDOGL1-R	59.72	ccaggagcttacaggtgtcc			
HSPA2-F	60.51	ccaccatcgaagaagtggac	109	HSPA2	4274 - 4684
HSPA2-R	60.09	ttggcacaaggacatttcaa			
MANEA-F	59.69	ttgctgtgaacctgaaatgg	106	MANEA	1820 - 2294
MANEA-R	59.88	agcaggccttcctaacacaa			
NARG2-F	59.88	aagcccacacttaagctcca	107	NARG2	6709-7209
NARG2-R	60	tttcctctccctcccctaaa			
NBN-F	60.1	aaccattccaatcggtgtgt	118	NBN	3825 - 4341
NBN-R	60	tccgacaagagacttgcatct			
PAQR3-F	60.59	ttaggaagggctcatgtcca	97	PAQR3	1356 - 1860
PAQR3-R	59.39	cacttgccttgattaggcact			
SKP2-F	60.12	catcaaatgccgactgacac	104	SKP2	1262 - 1518
SKP2-R	59.93	attgggcttcctgcctattt			
ZNF643-F	59.97	ggaaagcctttagccagagaa	90	ZNF643	1408 - 1934
ZNF643-R	58.57	ctttcccacaatgactgcat			
BTG2-F	59.83	agcctcatggtctcatgctt	103	BTG2	2178 - 2551
BTG2-R	60.06	agacaggcctgctcaacagt			
C12ORF5-F	60.52	ttgagcctctgaagggagtg	111	C12orf5	869 - 1286
C12ORF5-R	58.44	atgtggctatggttccactg			
CDKN1A-F	60.02	ttagcagcggaacaaggagt	120	CDKN1A	1555 - 2027
CDKN1A-R	59.32	tcaacgttagtgccaggaaa			
FAS-F	59.81	tgccaagaagggaaggagta	110	FAS	591 - 1045
FAS-R	60.37	cgggtgcagtttatttccac			
GADD45A-F	59.46	ggtgatggcatctgaatgaa	124	GADD45A	774 - 1156
GADD45A-R	60.11	cccttggcatcagtttctgt			
GDF15-F	60.16	gaggtgcaagtgaccatgtg	87	GDF15	433 - 944
GDF15-R	60.56	gtgcaggctcgtcttgatct			

IGFBP3-F	59.92	cagagactcgagcacagcac	98	IGFBP3	901-1401
IGFBP3-R	59.88	gccgcctaagtcacaaagtc			
LRDD-F	59.21	agagcaacctgctgagtgtg	122	LRDD	2380 - 2789
LRDD-R	60.68	atccagatcatcccggaact			
MDM2-F	59.95	cggaaagatggagcaagaag	116	MDM2	15 - 320
MDM2-R	59.61	gcgctcgtacgcactaatc			
SESN1-F	60.01	gttcccagcaccaaagttgt	82	SESN1	2237 - 2579
SESN1-R	59.88	aggcagaggcagagagactg			
TNFRSF10B-F	60.12	atgttggtcaggctggtctc	76	TNFRSF10B	3327 - 3874
TNFRSF10B-R	59.2	gcctgtaatcccagcacttt			
p53-F	60.39	tcaagacagaagggcctgac	118	p53	1352-1852
p53-R	59.94	caagggttcaaagacccaaa			
GAPDH-F	59.96	acccagaagactgtggatgg	125	GAPDH	546-1040
GAPDH-R	59.8	ttcagctcagggatgacctt			

Supplementary References

1. Webb MR, Ebeler SE. A gel electrophoresis assay for the simultaneous determination of topoisomerase I inhibition and DNA intercalation. Anal Biochem 2003;321:22-30.

2. Graves DE. Drug-DNA interactions. Methods Mol Biol 2001;95:161-9.

3. Jenkins TC. Optical Absorbance and Fluorescence Techniques for Measuring DNA-Drug Interactions. In: Fox KR, editor. Methods in Molecular Biology, Drug-DNA Interaction Protocols. Springer; 1997. vol 90, p.195-218.

4. Kiviharju-af Hällström, TM et al. Human prostate epithelium lacks Wee1A-mediated DNA damage-induced checkpoint enforcement. Proc. Natl. Acad. Sci. *USA* 2007;104: 7211-7216.

5. Sohn TA. Bansal R. Su GH. Murphy KM. & Kern SE. High-throughput measurement of the Tp53 response to anticancer drugs and random compounds using a stably integrated Tp53-responsive luciferase reporter. Carcinogenesis 2002; 23:949-957.

6. Gurova KV. et al. Small molecules that reactivate p53 in renal cell carcinoma reveal a NF-kappaB-dependent mechanism of p53 suppression in tumors. Proc. Natl. Acad. Sci. USA 2005;102:17448-17453.

7. Sun XX. Dai MS. & Lu H. Mycophenolic acid activation of p53 requires ribosomal proteins L5 and L11. J. Biol. Chem. 2008; 283:12387-12392.
