# Supplementary figures and images for: Identification of Novel p53 Pathway Activating Small-Molecule Compounds Reveals Unexpected Similarities with Known Therapeutic Agents
Source: PLoS One. 2010 Sep 27;5(9):e12996. doi: 10.1371/journal.pone.0012996 (PMC2946317; doi:10.1371/journal.pone.0012996)

Figure S1.

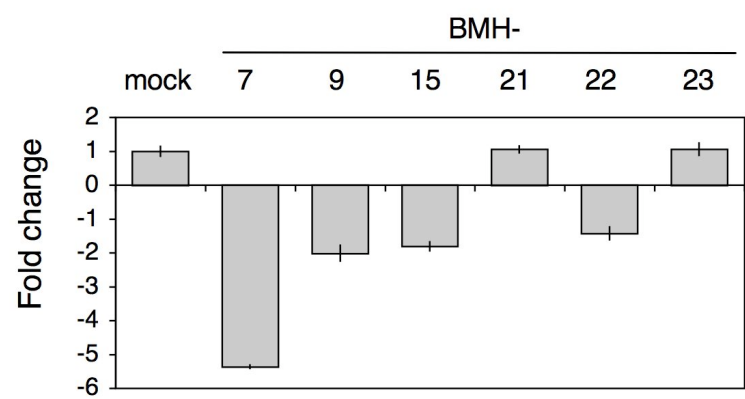

Supplement: Figure S1 — qPCR analysis of TP53 mRNA expression. MCF-7 cells were incubated with BMH-7, -9 (10 µM), BMH-15, -22, -23 (5 µM), and BMH-21 (0.5 µM) for 6 h, and control cultures were mock-treated with DMSO (n = 4). Total RNA was isolated and qPCR performed for TP53. The values were normalized according to GAPDH. Error bars represent SD. (0.15 MB PDF) [file pone.0012996.s004.pdf]

Figure S2.

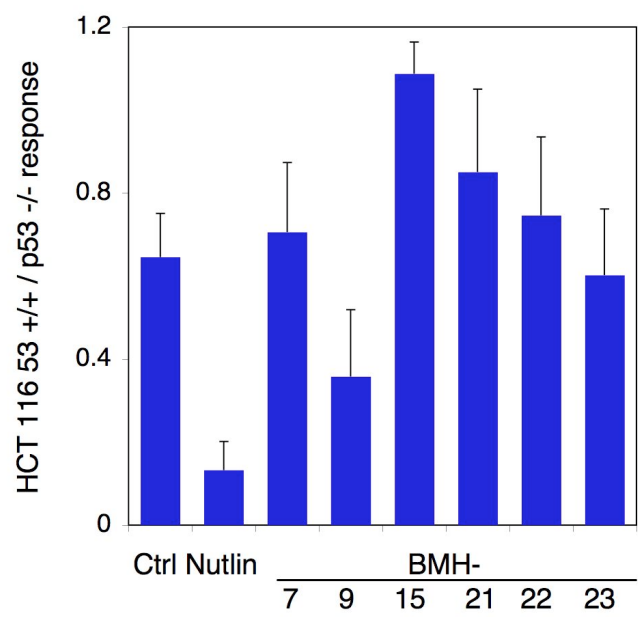

Supplement: Figure S2 — p53 dependency of cell viability. Lead compound effect on p53 isogenic HCT116 cells. HCT116 p53+/+ and p53−/− cells were cultured in the presence of BMH-7, -9, -15, -22, -23 (5 µM), BMH-21 (0.5 µM), and nutlin-3 (5 µM) for 72 h followed by counting of the cells. The relative viability response as adjusted to controls in the p53+/+ as compared to p53−/− cells is shown. Error bars represent SE. (0.16 MB PDF) [file pone.0012996.s005.pdf]

Figure S3.

**A**

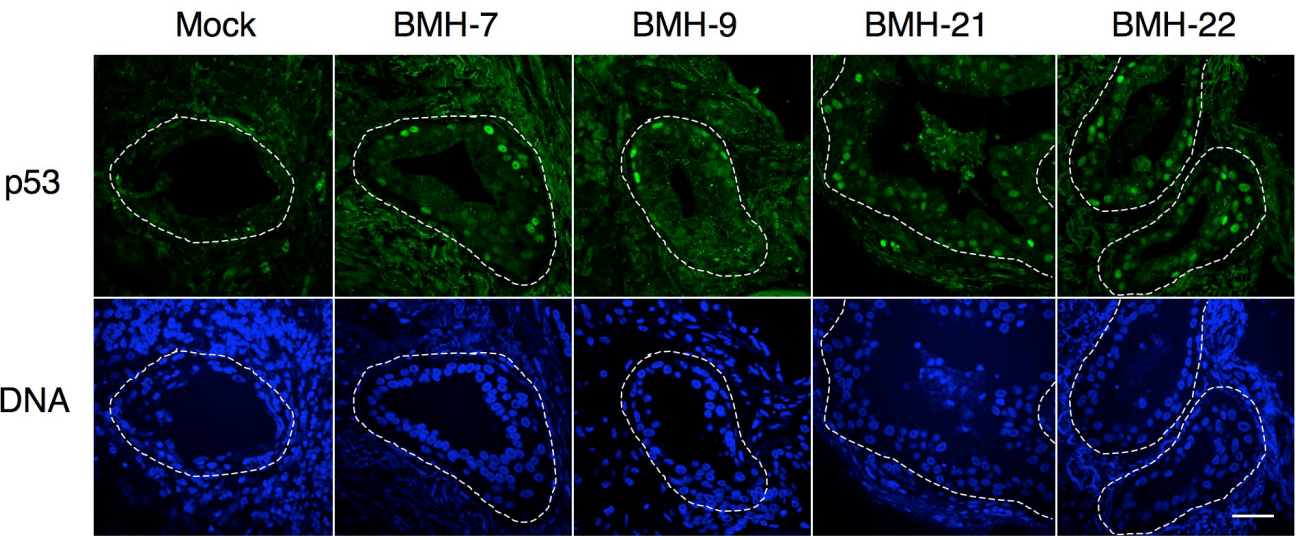

**B**

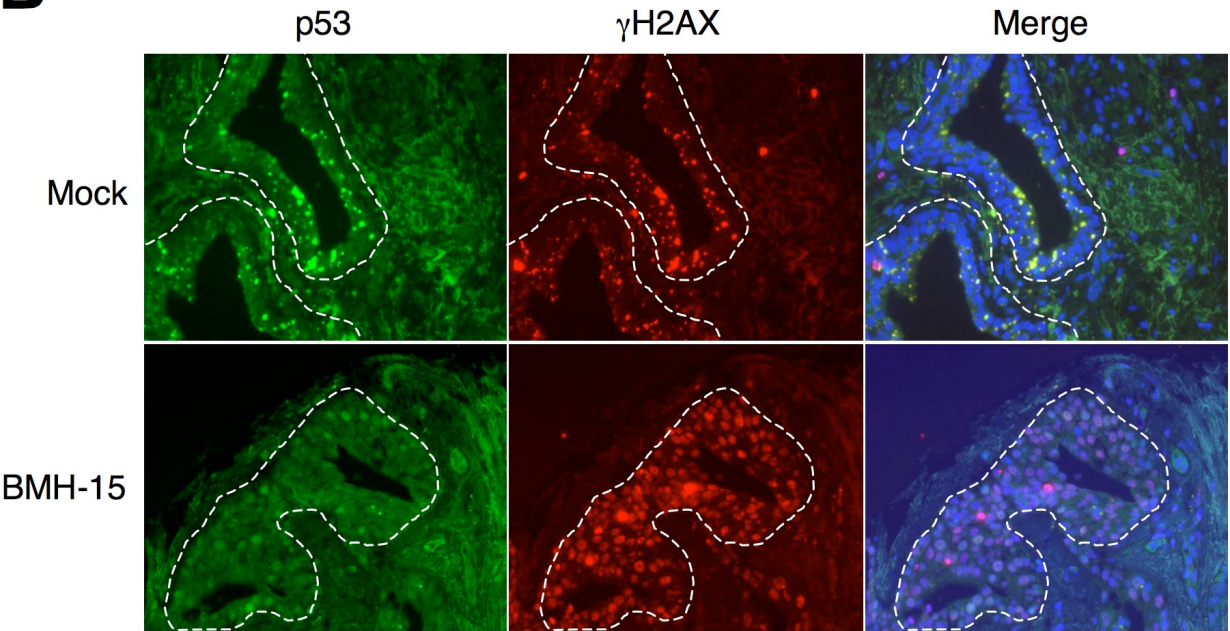

Supplement: Figure S3 — Human ex vivo prostate tissue. Fresh prostate tissues were obtained from radical prostatectomies, and the sections were incubated with BMH-7 (20 µM), BMH-9 (20 µM), BMH-15 (20 µM), BMH-21 (2 µM), and BMH-22 (20 µM) for 24 h, fixed and stained for p53 and DNA, and in (B) also for gamma-H2AX. Images were captured using confocal microscopy (A) or wide-field microscopy (B). Prostate glands are indicated by white dashed lines. Scale bar, 50 µm. (0.53 MB PDF) [file pone.0012996.s006.pdf]

**Figure S4.**

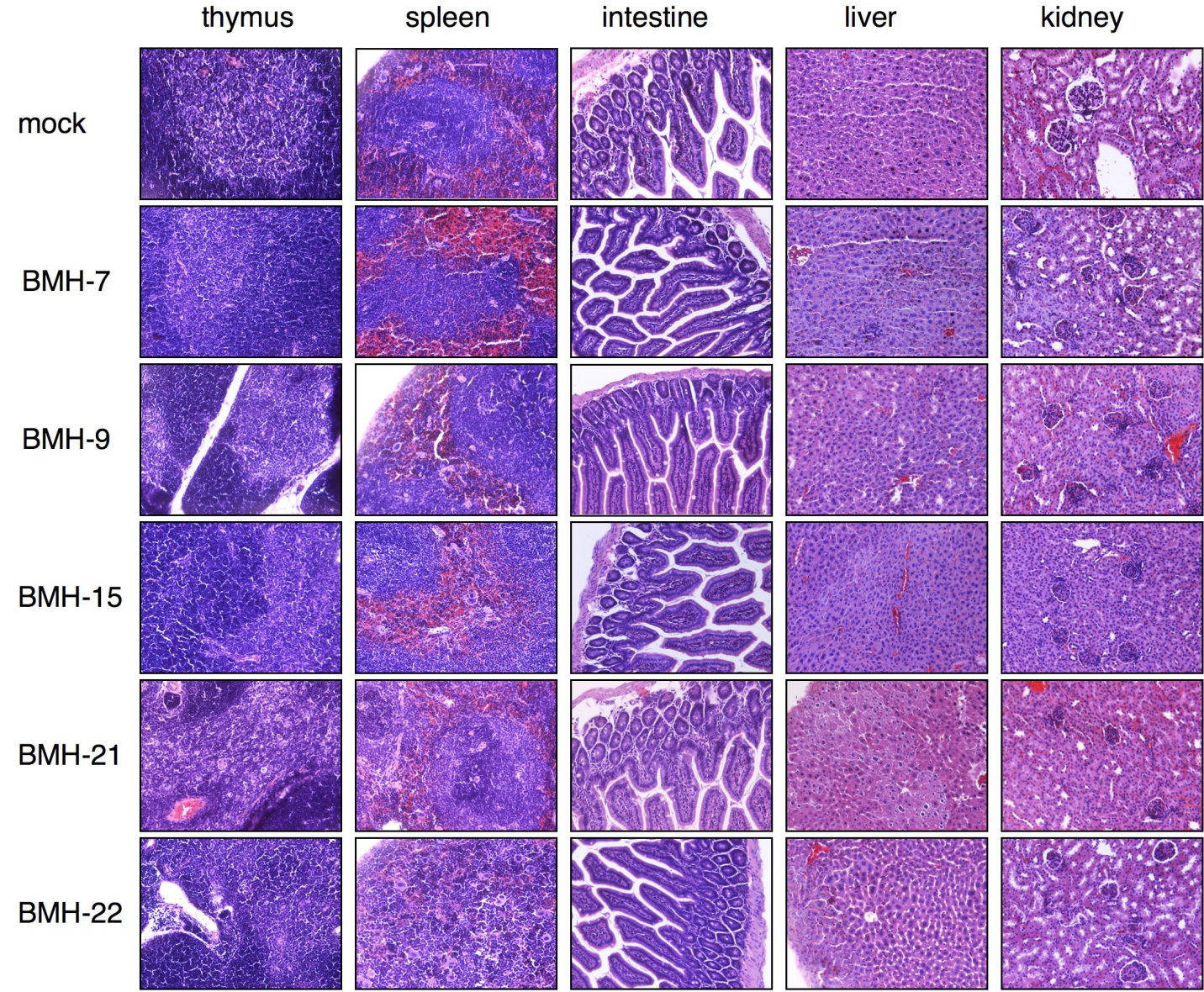

Supplement: Figure S4 — In vivo toxicity. Mice were injected intraperitoneally with BMH-7, -9, -15, -22 (20 mg/kg), and BMH-21 (2 mg/kg) three times a week for three weeks in 30 µl DMSO. Control animals received only the DMSO vehicle. The mice were sacrificed and organs (thymus, spleen, intestine, liver and kidney) were collected for histological examination. No acute or chronic toxicities were observed based on the histological hematoxylin-eosin analyses. Similarly, there were no changes in the weight curves of mice undergoing the above treatment regimen (N = 2 for each treatment group) (data not shown). (1.16 MB PDF) [file pone.0012996.s007.pdf]

Figure S5.

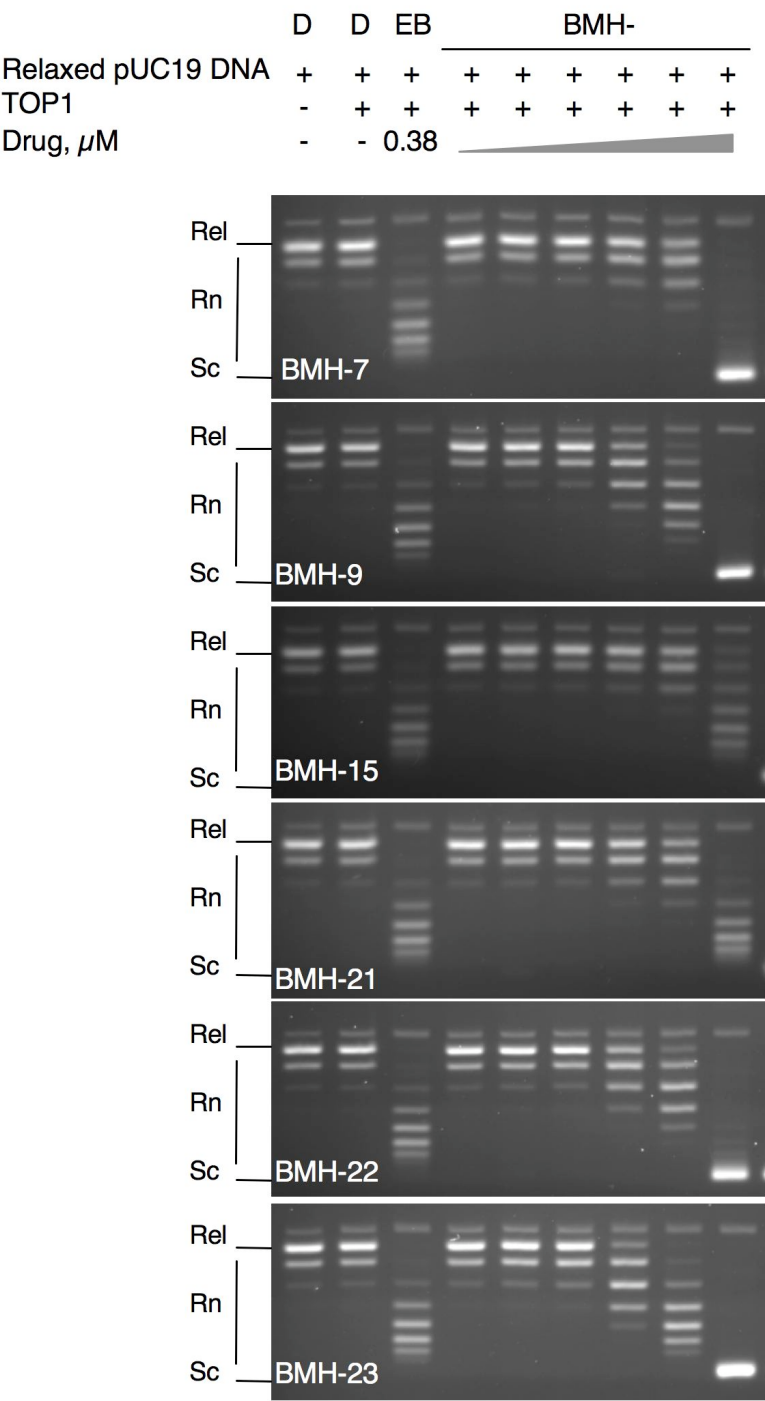

Supplement: Figure S5 — DNA unwinding. TOP1 (2 U) was added to plasmid DNA to allow full relaxation of the plasmid (Rel). Subsequently, an excess of TOP1 (20 U) and increasing amounts of compounds (BMH-7, -9, -15, -22, -23, 0.01-5 µM; BMH-21, 0.001-0.5 µM) were added and incubated for further 1 h at 37°C. The reaction was quenched and the samples were analyzed by agarose gel electrophoresis. Note appearance of DNA topomers (Rn) and supercoiled DNA (Sc) due to intercalation. D, DMSO control, EB ethidium bromide. (0.22 MB PDF) [file pone.0012996.s008.pdf]
