# Supplementary material for: Identification of Novel p53 Pathway Activating Small-Molecule Compounds Reveals Unexpected Similarities with Known Therapeutic Agents
Source: PLoS One. 2010 Sep 27;5(9):e12996. doi: 10.1371/journal.pone.0012996 (PMC2946317; doi:10.1371/journal.pone.0012996)
Supplement: Table S1 — p53 activating drugs in the Spectrum Collection. (0.06 MB PDF) [file pone.0012996.s009.pdf]

Supplementary Table S1. p53 activating drugs in the Spectrum Collection

Name	Drug class	Mechanism	p53 stabilization*	Links to p53
acrisorcin	antifungal	DNA intercalation	5.3	Refs. 5, 6
aminacrine	antiseptic	DNA intercalation	5.4	Refs. 5, 6
quinacrine HCl	antimalarial, antihelmintic	DNA intercalation	4.5	Refs. 5, 6
acriflavinium HCl	antiseptic	DNA intercalation	6.0	None
hycanthone	antihelmintic	DNA intercalation	2.8	None
10-OHcamptothecin	antineoplastic	topo I inhibitor	5.2	Yes, extensive
teniposide	antineoplastic	topo II inhibitor	4.4	Yes, extensive
etoposide	antineoplastic	topo II inhibitor	2.0	Yes, extensive
aclacinomycin A1	antibiotic	anthracyclin, topo II inhibitor	5.8	None
rutilantin	antibiotic	anthracyclin	5.4	None
mycophenolic acid	immunosuppressant	purine biosynthesis inhibitor	3.4	Ref. 7

* Fold change of mean p53 protein staining intensity as compared to mock determined by quantitative image analysis
