# Supplementary material for: Identification of Novel p53 Pathway Activating Small-Molecule Compounds Reveals Unexpected Similarities with Known Therapeutic Agents
Source: PLoS One. 2010 Sep 27;5(9):e12996. doi: 10.1371/journal.pone.0012996 (PMC2946317; doi:10.1371/journal.pone.0012996)
Supplement: Table S2 — Lipinsky rule of five. (0.06 MB PDF) [file pone.0012996.s010.pdf]

Supplementary Table S2. Lipinsky rule of five

	BMH-7	BMH-9	BMH-15	BMH-21	BMH-22	BMH-23
MW (<500)	347.4	329.5	381.49	360.4	251.3	237.3
CLOGP (<5)	2.281±0.69	5.319±0.4	2.880±0.9	1.57±0.8	3.197±0.8	2.736±0.8
H-bond acceptors (<10)	6	5	6	6	3	3
H-bond donors (<5)	1	1	1	1	2	2
