# Supplementary material for: Identification of Novel p53 Pathway Activating Small-Molecule Compounds Reveals Unexpected Similarities with Known Therapeutic Agents
Source: PLoS One. 2010 Sep 27;5(9):e12996. doi: 10.1371/journal.pone.0012996 (PMC2946317; doi:10.1371/journal.pone.0012996)
Supplement: Table S3 — In vitro normal and melanoma cell line viability responses. (0.07 MB PDF) [file pone.0012996.s011.pdf]

Supplementary Table S3. In vitro normal and melanoma cell line viability responses*

	BMH-7	BMH-9	BMH-21	BMH-22	Nutlin
A375	3.0±0.8	6.1±2.0	0.7±1.1	4.6±1.4	7.2±2.4
Fibroblast¶	18.1±2.6	15.7±5.0	1.9±3.6	11.5±5.7	20.6±6.9
Melanocyte#	32.4±1.1	≥40	≥40	32.2±0.7	≥40
HIMEC§	19.6±2.8	19.6±1.6	2.7±5.1	14.5±2.0	10.7±10.7
All normal (average)	23.4±7.9	25.1±13	14.8±21.8	19.4±11.2	23.8±14.9

*IC50 μM±SD, ¶WS-1 human diploid fibroblasts, #primary human melanocytes, §human intestinal microvascular endothelial cells
