# Supplementary material for: Identification of Novel p53 Pathway Activating Small-Molecule Compounds Reveals Unexpected Similarities with Known Therapeutic Agents
Source: PLoS One. 2010 Sep 27;5(9):e12996. doi: 10.1371/journal.pone.0012996 (PMC2946317; doi:10.1371/journal.pone.0012996)
Supplement: Table S4 — DNA intercalation of the compounds by UV-VIS. (0.06 MB PDF) [file pone.0012996.s012.pdf]

Supplementary Table S4. DNA intercalation of the compounds by UV-VIS

	**BMH-7**	**BMH-9**	**BMH-15**	**BMH-21**	**BMH-22**	**BMH-23**
Bathochromic shift (nm)	2	0	7	4	6	8
Hypochromic shift (%)	10.2	30.5	13.5	25.3	30.7	42.0
