# Supplementary material for: Identification of Novel p53 Pathway Activating Small-Molecule Compounds Reveals Unexpected Similarities with Known Therapeutic Agents
Source: PLoS One. 2010 Sep 27;5(9):e12996. doi: 10.1371/journal.pone.0012996 (PMC2946317; doi:10.1371/journal.pone.0012996)
Supplement: Table S5 — GO categories of transcriptional targets. (0.06 MB PDF) [file pone.0012996.s013.pdf]

Supplementary Table S5. GO categories of transcriptional targets*.

**BMH-7**	***P***	**fold**	**BMH-9**	***P***	**fold**
sterol/steroid metabolism	1.56E-08	4.9	DNA damage response signaling	6.02E-07	6.3
ribosome biogenesis and assembly	2.02E-05	3.7	M-phase	2.15E-26	5.7
protein-RNA complex assembly	7.40E-06	3.5	methylation	2.44E-04	4.6
mitosis	2.86E-06	2.7	cell cycle	1.11E-27	4.6
lipid biosynthetic process	3.04E-06	2.5	meiosis	4.81E-04	3.9
			DNA repair	1.75E-11	3.5
			interphase	2.82E-04	3.3
			cell cycle regulation	2.66E-15	3
			ribosome biogenesis and assembly	0.016	2.6
**BMH-15**	***P***	**fold**	**BMH-21**	***P***	**fold**
sterol/steroid metabolism	3.49E-09	5.4	DNA damage response signaling	1.32E-06	5.8
translational initiation	1.22E-04	5.1	RNA splicing	0.00152	3.3
protein-RNA complex assembly	1.26E-04	3.2	interphase	1.62E-04	3.3
lipid biosynthetic process	8.88E-06	2.5	M-phase	1.60E-09	3.3
			cell cycle	7.43E-11	2.9
			DNA repair	6.52E-06	2.5
**BMH-22**	***P***	**fold**	**BMH-23**	***P***	**fold**
DNA damage response signaling	5.70E-05	4.9	DNA damage response signaling	8.32E-07	6.1
meiosis	8.67E-04	3.6	M-phase	1.24E-10	3.5
RNA splicing	0.00154	3.3	cell cycle	1.63E-11	3
anterior/posterior pattern formation	0.00443	3.1	interphase	0.00403	2.8
protein-RNA complex assembly	3.00E-04	3	DNA repair	9.13E-07	2.7
unfolded protein response	0.00411	2.7			
DNA repair	2.37E-06	2.6			
Golgi vesicle transport	0.00496	2.5			

*Level BP4 $P < 0.05$, fold enrichment >2.5, >10 targets/category
