# Supplementary material for: Identification of Novel p53 Pathway Activating Small-Molecule Compounds Reveals Unexpected Similarities with Known Therapeutic Agents
Source: PLoS One. 2010 Sep 27;5(9):e12996. doi: 10.1371/journal.pone.0012996 (PMC2946317; doi:10.1371/journal.pone.0012996)
Supplement: Table S6 — KEGG pathways of transcriptional targets. (0.07 MB PDF) [file pone.0012996.s014.pdf]

**Supplementary Table S6.** KEGG pathways of transcriptional targets*.

| **BMH-7** | ***P*** | **fold** | **BMH-9** | ***P*** | **fold** |
|---|---|---|---|---|---|
| biosynthesis of steroids | 2.58E-12 | 11 | cell cycle | 9.74E-10 | 4.3 |
| cell cycle | 1.44E-05 | 3.1 | p53 signaling pathway | 2.84E-09 | 5.5 |
| p53 signaling pathway | 0.00125 | 3.1 | ubiquitin mediated proteolysis | 2.51E-04 | 2.7 |
| ubiquitin mediated proteolysis | 0.0144 | 2 | colorectal cancer | 0.00321 | 2.8 |
| colorectal cancer | 0.0477 | 2 | | | |
| **BMH-15** | ***P*** | **fold** | **BMH-21** | ***P*** | **fold** |
| biosynthesis of steroids | 4.74E-14 | 12.4 | cell cycle | 8.58E-06 | 3 |
| ubiquitin mediated proteolysis | 0.00975 | 2.1 | metabolism of xenobiotics by CYP450 | 8.12E-05 | 3.4 |
| cell cycle | 0.00280 | 2.4 | porphyrin and chorophyll metabolism | 1.99E-04 | 4.1 |
| | | | p53 signaling pathway | 0.00114 | 3 |
| | | | starch and glucose metabolism | 0.00178 | 2.7 |
| | | | gap junction | 0.00278 | 2.4 |
| | | | long-term potentiation | 0.00804 | 2.6 |
| | | | ubiquitin mediated proteolysis | 0.00943 | 2 |
| | | | GnRH signaling pathway | 0.0168 | 2.1 |
| **BMH-22** | ***P*** | **fold** | **BMH-23** | ***P*** | **fold** |
| p53 signaling pathway | 1.82E-05 | 3.9 | p53 signaling pathway | 7.98E-09 | 5.2 |
| cell cycle | 1.83E-04 | 2.8 | cell cycle | 1.87E-08 | 3.9 |
| ubiquitin mediated proteolysis | 0.00258 | 2.3 | ubiquitin mediated proteolysis | 5.50E-05 | 2.8 |
| antigen processing and presentation | 0.00484 | 2.6 | | | |

* *P*< 0.05, fold enrichment >2.0, >10 targets/category
