# Supplementary material for: Identification of Novel p53 Pathway Activating Small-Molecule Compounds Reveals Unexpected Similarities with Known Therapeutic Agents
Source: PLoS One. 2010 Sep 27;5(9):e12996. doi: 10.1371/journal.pone.0012996 (PMC2946317; doi:10.1371/journal.pone.0012996)
Supplement: Table S7 — Summary of top-ranking connectivities. (0.06 MB PDF) [file pone.0012996.s015.pdf]

Supplementary Table S7. Summary of top-ranking connectivities.

Drug class	No of instances	% instances	No of drugs	p53 activation	Drugs
quinoline	23	19.2	8	1	quinostatin, mepacrine, pyrvinium, primaquine, mefloquine, clioquinol, amodiaquine, ethaverine
topoisomerase I/II inhibitor	20	16.7	8	8	daunorubicin, irinotecan, ellipticine, mitoxantrone, etoposide, camptothecin, doxorubicin, harmine
phenothiazine	12	10.0	6	-	prochlorperazine, thioridazine, trifluoperazine, fluphenazine, promethazine, perphenazine
glycoside	7	5.8	4	2	digoxin, ouabain, quercetin, proscillaridin
antihistamine	6	5.0	2	1	terfenadine, astemizole
α-1 receptor antagonist	6	5.0	2	-	prazosin, phenoxybenzamine
PI3K inhibitor	5	4.2	2	-	LY294002, wortmannin
DNA intercalator	5	4.2	1	-	hycanthone
dopamine agonist/serotonin antagonist	4	3.3	1	-	metergoline
phytosterol	3	2.5	1	1	resveratrol
glucocorticoid	3	2.5	1	-	mometasone
NMDA antagonist	2	1.7	1	1	dizocilpine
Ca2+ blocker	2	1.7	1	-	suloctidil
other	22	18.3	21	4	
Total	120	100.0	59	18	
